# Teuvincenone F Suppresses LPS-Induced Inflammation and NLRP3 Inflammasome Activation by Attenuating NEMO Ubiquitination

**DOI:** 10.3389/fphar.2017.00565

**Published:** 2017-08-23

**Authors:** Xibao Zhao, Debing Pu, Zizhao Zhao, Huihui Zhu, Hongrui Li, Yaping Shen, Xingjie Zhang, Ruihan Zhang, Jianzhong Shen, Weilie Xiao, Weilin Chen

**Affiliations:** ^1^Department of Immunology, School of Medicine, Shenzhen University Shenzhen, China; ^2^Institute of Immunology, Department of Basic Medicine, Zhejiang University School of Medicine Hangzhou, China; ^3^Key Laboratory of Medicinal Chemistry for Natural Resource, Ministry of Education, School of Chemical Science and Technology, Yunnan University Kunming, China; ^4^State Key Laboratory of Phytochemistry and Plant Resources in West China, Kunming Institute of Botany, Chinese Academy of Sciences Kunming, China; ^5^Department of Drug Discovery and Development, Harrison School of Pharmacy, Auburn University Auburn, AL, United States

**Keywords:** teuvincenone F, inflammation, NLRP3 inflammasome, ubiquitination, NEMO

## Abstract

Inflammation causes many diseases that are serious threats to human health. However, the molecular mechanisms underlying regulation of inflammation and inflammasome activation are not fully understood which has delayed the discovery of new anti-inflammatory drugs of urgent clinic need. Here, we found that the natural compound Teuvincenone F, which was isolated and purified from the stems and leaves of *Premna szemaoensis*, could significantly inhibit lipopolysaccharide (LPS)–induced pro-inflammatory cytokines production and NLRP3 inflammasome activation. Our results showed that Teuvincenone F attenuated K63-linked ubiquitination of NF-κB-essential modulator (NEMO, also known as IKKγ) to suppress LPS-induced phosphorylation of NF-κB, and inhibited mRNA expression of IL-1β, IL-6, TNF-α, and NLRP3. In addition, we found that decreased NLRP3 expression by Teuvincenone F suppressed NLRP3 inflammasome activation and IL-1β/IL-18 maturation. *In vivo*, we revealed that Teuvincenone F treatment relieved LPS-induced inflammation. In conclusion, Teuvincenone F is a highly effective natural compound to suppress LPS-induced inflammation by attenuating K63-linked ubiquitination of NEMO, highlighting that Teuvincenone F may be a potential new anti-inflammatory drug for the treatment of inflammatory and NLRP3 inflammasome-driven diseases.

## Introduction

The type I transmembrane receptors Toll-like receptors (TLRs) play a crucial role in the innate immune and adaptive immunity system (Takeda and Akira, 2005; Akira et al., 2006; Fu et al., 2013). TLR4 is activated by lipopolysaccharide (LPS), the integral molecules within the outer membrane of gram-negative bacteria. Upon stimulation by LPS, TLR4 is recruited to lipid rafts and interacts with its adaptor molecules (Fu et al., 2014; Wei et al., 2016), leading to MyD88-dependent and MyD88-independent signaling pathways activation, resulting in activation of Nuclear factor κB (NF-κB) and Interferon Regulatory Factor 3 (IRF3) and cytokines production (Fitzgerald et al., 2003; Fu et al., 2014; Wei et al., 2016). Previous studies have demonstrated that the transcription factors of NF-κB family is activated in response to many stimulation, including environmental stresses, pro-inflammatory cytokines, and antigenic stimulation (Silverman and Maniatis, 2001; Dixit and Mak, 2002; Zhou et al., 2004). However, the excessive inflammation activation can result in many diseases, such as sepsis, a deadly immunological disorder, which is caused by excessive systemic immune response to invasive microbial pathogens (Zhao et al., 2015a).

NF-κB is a key transcription factor in many physiological process and cellular functions by regulating of immune development, immune responses, inflammatory responses and cancer (Mitchell et al., 2016). The classical NF-κB pathway activation involves in the IκB kinase (IKK) complex (Khoshnan et al., 2000). The IKK complex, which is composed of two catalytic subunits, IKKα and IKKβ, as well as the crucial regulatory subunit NEMO (Chen, 2005). IKKs can phosphorylate the IκB and promoted this NF-κB activation inhibitor for degradation via the ubiquitin-proteasome pathway (Chen, 2005; Krappmann and Scheidereit, 2005), resulting in translocation of NF-κB to the nucleus. In this NF-κB activation process, the regulatory subunit NEMO liner polyubiquitination is indispensable (Tang et al., 2003; Temmerman et al., 2006; Wu et al., 2006). Studies have revealed many molecular mechanisms of NEMO ubiquitination and deubiquitination in the past decades. The adaptor protein Bcl10 promotes NF-κB transcription factors activation through paracaspase and UBC13-dependent NEMO ubiquitination, this result reveal that UBC13 might be an E3 ligase for NEMO (Zhou et al., 2004). However, HSCARG and USP7 had an important function in suppressing NEMO polyubiquination, and then suppressing NF-κB activity, in this process, suggesting that USP7 was a deubiquitinase of NEMO (Li et al., 2014). Besides, c-IAP1 is also essential for NEMO ubiquitination and regulates the proper activation of the IKK signalsome complex by TNF-α activation (Tang et al., 2003). Thus, these studies suggested that inhibiting the NEMO ubiquitination might be an ideal way to cure excessive inflammation caused by infection.

Upon the cellular infection or stress, triggering the molecular platforms inflammasomes activated, promoted the proinflammatory cytokines maturation and secretion, such as IL-1β, to engage innate immune defenses (Schroder and Tschopp, 2010; Huang et al., 2015). Studies confirmed that several NOD-like receptors (NLRs) family members were capable to form inflammasomes in response to their specific stimulators. Currently, the NLRP3 inflammasome is the most studied inflammasome. The NLRP3 inflammasome is a cytosolic protein complex, it composed of NLRP3, caspase-1, and ASC, and assembled to form an activity protein complex in response to both microbial infection and endogenous “danger signal” (Martinon et al., 2009; Davis et al., 2011; Yan et al., 2015). Previous studies showed that several regulatory mechanisms could inhibit NLRP3 inflammasome activation. LRRFIP2 can inhibit NLRP3 inflammasome activation by recruiting the caspase-1 inhibitor Flightless-I (Jin et al., 2013). Dopamine signals inhibit NLRP3 inflammasome activation through DRD1 and suppress the transcription proinflammatory cytokines through DRD2 (Yan et al., 2015). Omega-3 fatty acids can inhibit NLRP3 and NLRP1b-dependent caspase-1 activation and IL-1β secretion (Yan et al., 2013). Previous study also showed that cell priming through multiple signaling receptors promoted NF-κB activation to induce NLRP3 expression, which is a critical checkpoint for NLRP3 activation (Bauernfeind et al., 2009). The specific role of priming for NLRP3 inflammasome activation was reflected by the fact that NLRP3 was a highly inducible gene in response to proinflammatory stimulation, such as LPS. Of interest, LPS priming enhanced neither the expression of NLRP1a nor that of NLRP1b, NLRC4, AIM2, caspase-1, or ASC (Bauernfeind et al., 2011). These data indicated that NLRP3 could be unique among the known inflammasome pathways in its specific requirement of a proinflammatory priming signal.

The genus *Premna* belongs to the family of *Verbenaceae*. There are about 200 species distributed throughout the tropical and subtropical regions of the world (Kadereit, 2004), of which some are well-known for their medicinal properties and have been extensively used for traditional medicine, especially to treat pyogenic infections, trauma, fracture, dysentery, hemorrhoids, rheumatic arthritis in China (Pei and Chen, 1982) and diarrhea, stomach, and hepatic disorders in Indian (Devi et al., 2004). *Premna szemaoensis Pei* is mainly distributed in the south of Yunnan province of China. Local residents used leaves of the tree to cure injuries and fracture (Wu, 1977). We isolated and purified a series of compounds from the stems and leaves of *P. szemaoensis*, and screened the effective anti-inflammatory compound, as a potential drug used to cure the diseases caused by inflammation. We found that Teuvincenone F could significantly inhibit LPS–induced proinflammatory cytokines production and NLRP3 inflammasome activation. Teuvincenone F is a diterpenoid compound with a molecular formula of C_20_H_18_O_5_,which was first separated from the roots of *Teucrium fruticans* (Caudvado et al., 1992), In the present study, the chemical structure of Teuvincenone F was elucidated by extensive spectroscopic means, and further determined by single-crystal X-ray crystallography for the first time. However, the pharmacological function of Teuvincenone F *in vitro* and *in vivo* is still elusive.

Here, we demonstrated that Teuvincenone F might be a negative regulator of LPS-induced inflammation via attenuating K63-ubiquitination of NEMO, highlighting its potential as a new anti-inflammatory drug for the treatment of inflammatory and NLRP3 inflammasome-driven diseases.

## Materials and methods

### Mice and cell culture

Wild type C57BL/6 mice (6–8 weeks), weighing 16–25 g, were purchased from Joint Ventures Sipper BK Experimental Animals. Mice were housed in individual and pathogen-free condition. All animal experiments were conducted according to the National Institute of Health Guide for the Care and Use of Laboratory Animals, and the experiment protocols were approval by the Zhejiang University, Hangzhou. Mouse peritoneal macrophages were collected from mice, as described previously (Zhao et al., 2016). THP-1 cells were from ATCC and cultured in 1,640 medium supplemented with 10% fetal bovine serum.

### Antibodies and reagents

Antibodies against Caspase-1 (p10) (Cat. No: AG-20B-0044) and ASC (Cat. No: AG-25B-0006) were purchased from Adipogen International. Antibody against NEMO (also named IKKγ, Cat. No: sc-8330) was purchased from Santa Cruz Biotechnology. Antibodies specific to IL-1β (Cat. No: 12242), NLRP3 (Cat. No: 15101), NF-κB Pathway Sampler Kit (Cat. No: 9936), and Phospho-MAPK Family Antibody Sampler Kit (Cat. No: 9910) were obtained from Cell Signaling Technology. β-actin antibody (Cat. No: EM21002) was purchased from HuaAn Biotechnology. Reagents used in this study included the following: ATP (Cat. No: A7699), DMSO (Cat. No: D5879), and LPS (Cat. No: L3024) were obtained from Sigma-Aldrich. Nigericin was obtained from InvivoGen Biotechnology, PYR-41 (Cat. No: S7129) and PR-619 (Cat. No: S7130) were purchased from Selleck.

### Extraction and isolation of natural compounds

The air-dried and powdered aerial parts of *P*. *szemaoensis* (10 kg) were extracted with 70% aqueous acetone (40 L) four times (2 days each time) at room temperature. The extract was evaporated under reduced pressure at 40°C and then partitioned between n-butanol and H2O. The n-butyl alcohol soluble fraction (600 g) was chromatographed on silica gel CC (2.5 kg, 100–200 mesh), eluted with a CHCl3-Me2CO gradient system (1:0 to 0:1) to obtain fractions A-E. The fractions were then decolorized using MCI gel and eluted with 95% MeOH-H2O, respectively.

Fraction B (33 g) was chromatographed on silica gel CC (200–300 mesh), eluted with a CHCl3-MeOH gradient (150:1-1:1), to yield fractions B1-B5. Fraction B1 was purified by repeated silica gel CC (petroleum ether-Me2CO gradient, 12:1-0:1) to yield compounds 4 (3.4 g) and 5 (1.5 g). Compounds 6 (8.6 mg), 16 (3.6 mg), and 17 (8.3 mg) was isolated from fraction B2 by repeated silica gel CC (CHCl3-Acetone gradient, 15:1-1:1) and then by semi-preparative HPLC. Fraction B3 was separated by Sephadex LH-20 (MeOH), then Compound 1 (93.2 mg) was crystallized from the fraction and Compounds 7 (10.7 mg), 8 (4.2 mg), 9 (6.4 mg), and 10 (3.8 mg) were isolated by HPLC (42% MeOH-H2O). B4 was purified by RP-18 CC (MeOH-H2O gradient, 30–100%), followed by semi-preparative HPLC (45% MeOH-H2O), to obtain compounds 12 (10.1 mg), 13 (15.5 mg) and 14 (5.3 mg). Compounds 19 (4.1 mg) and 20 (3.5 mg) were purified from fraction B5 by Sephadex LH-20 CC (MeOH) and further by semi-preparative HPLC.

Fraction C (120 g) was subjected to RP-18 CC (MeOH−H2O, 10%−100%) to give fractions C1-C4. Fraction D4 was separated by Sephadex LH-20 eluted with MeOH, followed by semi-preparative HPLC to yield compounds 15 (11.0 mg) and 18 (17.2 mg). Fraction C3 was chromatographed on repeated silica gel CC (200–300 mesh), eluted with CHCl3-MeOH (gradient system: 120:1-1:1), to yield fractions C31-C34. Then, compound 11 (8.7 mg) was purified by semi-preparative HPLC from C32. Fraction C4 was separated by silica gel CC (200–300 mesh) eluted with CHCl3-MeOH (50:1-1:2), followed by HPLC (27% MeOH-H2O) to afford compounds 2 (18.3 mg) and 3 (15.5 mg).

### X-ray crystal structure analysis

Crystals of Teuvincenone F were obtained in MeOH. Crystallographic data were collected at 100 K on a Bruker APEX DUO diffractometer equipped with an APEX II CCD using Cu Kα radiation. Cell refinement and data reduction were performed with Bruker SAINT. The structures were solved by direct methods using SHELXS-97 (Sheldrick and Schneider, 1997). Refinements were performed with SHELXL-97 using full-matrix least-squares, with anisotropic displacement parameters for all the non-hydrogen atoms. The non-hydrogen atoms were refined anisotropically, and hydrogen atoms were fixed at calculated positions. Molecular graphics were computed with PLATON (Spek, 2015). Crystallographic data for the structure reported have been deposited at the Cambridge Crystallographic Data Center as supplementary publications no. CCDC 1547843 for Teuvincenone F. Copies of the data can be obtained free of charge on application to CCDC, 12 Union Road, Cambridge CB 1EZ, U.K. [fax: int. +44(0) (1223) 336 033); e-mail: deposit@ccdc.cam.ac.uk].

### Quantitative PCR and luciferase activity assay

Total cellular RNA was extracted by Trizol reagent. Gene-specific primer sequences were described in Table S1. For luciferase activity assay, RAW 264.7 cells were co-transfected with 90 ng of NF-κB luciferase reporter plasmid, 10 ng of the Renilla luciferase construct phRL-TK (Promega), and transfection of plasmids using JetPEI polyplus, following the manufacturer's instructions protocol. Twenty-four hours after transfection, cells were pretreated with DMSO or Teuvincenone F (25 μM) for 2 h, followed by stimulation with LPS (100 ng/ml) for indicated hours. Luciferase activity assays used the dual-specific luciferase assay kit (Promega) following the manufacturer's instructions.

### Immunoblot analysis and ubiquitination assay

Cells were pretreated with DMSO or Teuvincenone F (25 μM) for 2 h, followed by stimulation with LPS (100 ng/ml) for indicated times. For immunoblot assay, cells were lysed with 1× cell lysis buffer containing protease inhibitor mixture. After centrifugation for 5 min at 12,000 g, supernatants were collected and boiled with 1% (wt/vol) SDS sample buffer. For ubiquitination assay, cells were lysed in immunoprecipitation buffer (with 1% SDS) containing protease inhibitor mixture and boiled for 5 min at 95°. Supernatant was collected and diluted 10-fold in immunoprecipitation buffer, following immunoprecipitation with NEMO antibody for 2 h, and then incubated with protein A/G Plus-Agarose Immunoprecipitation reagent (Santa Cruz Biotechnology) for 6 h, beads was washed five times with immunoprecipitation buffer. Protein samples were boiled with SDS sample buffer 5 min and equal amounts of protein were used to 8%-12% SDS-PAGE and transferred into nitrocellulose membranes, immunoblot used specific antibodies, as described previously (Zhao et al., 2015b).

### Cell preparation and stimulation

For inducing IL-1β secretion, 1 × 10^6^ macrophages were seeded in 12 well sterile plate overnight and the culture medium was changed to opti-MEM in the next morning, cells were pretreated with DMSO or Teuvincenone F (25 μM) for 2 h, then stimulated with LPS (300 ng/ml) for 3 h. Next, cells were treated with ATP (1 mM) or nigericin (10 μM) for 45 min. The protein of collection from cell culture supernatants, as described previously (Jin et al., 2013). Cell total protein and precipitated supernatants protein were measured by immunoblotting.

### ELISA and MTT assay

For ELISA assay, mouse IL-1β, IL-6, TNF-α, IL-18, and human IL-1β, IL-6, TNF-α ELISA assays were performed according to the manufacturer's instructions (eBioscience Biotechnology). Mouse primary macrophages and THP-1 cells were plated in 96-well plates overnight, cells were treated with DMSO or different concentrations Teuvincenone F (0–25 μM) for 12 h and MTT assay was used to measure cell viability according to the manufacturer's instructions (Beyotime Biotechnology).

### Animal experiments

To induce cytokine secretion *in vivo*, mice (6–8 weeks) were pretreated with DMSO or Teuvincenone F (5 mg/kg, intraperitoneal injection) for 30 min, and challenged with PBS or LPS (10 mg/kg, intraperitoneal injection). After 2 h, the serum samples were collected and the cytokines were measured. For bronchoalveolar lavage assay, the trachea was exposed through a midline incision and cannulated with a sterile needle. Bronchial alveolar lavage fluid was obtained by washing with 1 ml EDTA/PBS. After centrifugation, the supernatants were used to measure cytokines by ELISA.

### Statistical analysis

All data are presented as the mean ± *SD*, and at least three independent experiments performed in duplicate. GraphPad Prism 5.0 was used for plotting data. Treatment effects were statistically analyzed by Student's *t*-test, and *p* < 0.05 was considered to be statistically significant (^*^p < 0.05; ^**^ *p* < 0.01; ^***^ *p* < 0.001).

## Results

### Screening potent natural anti-inflammatory compounds

**Figure 1:**
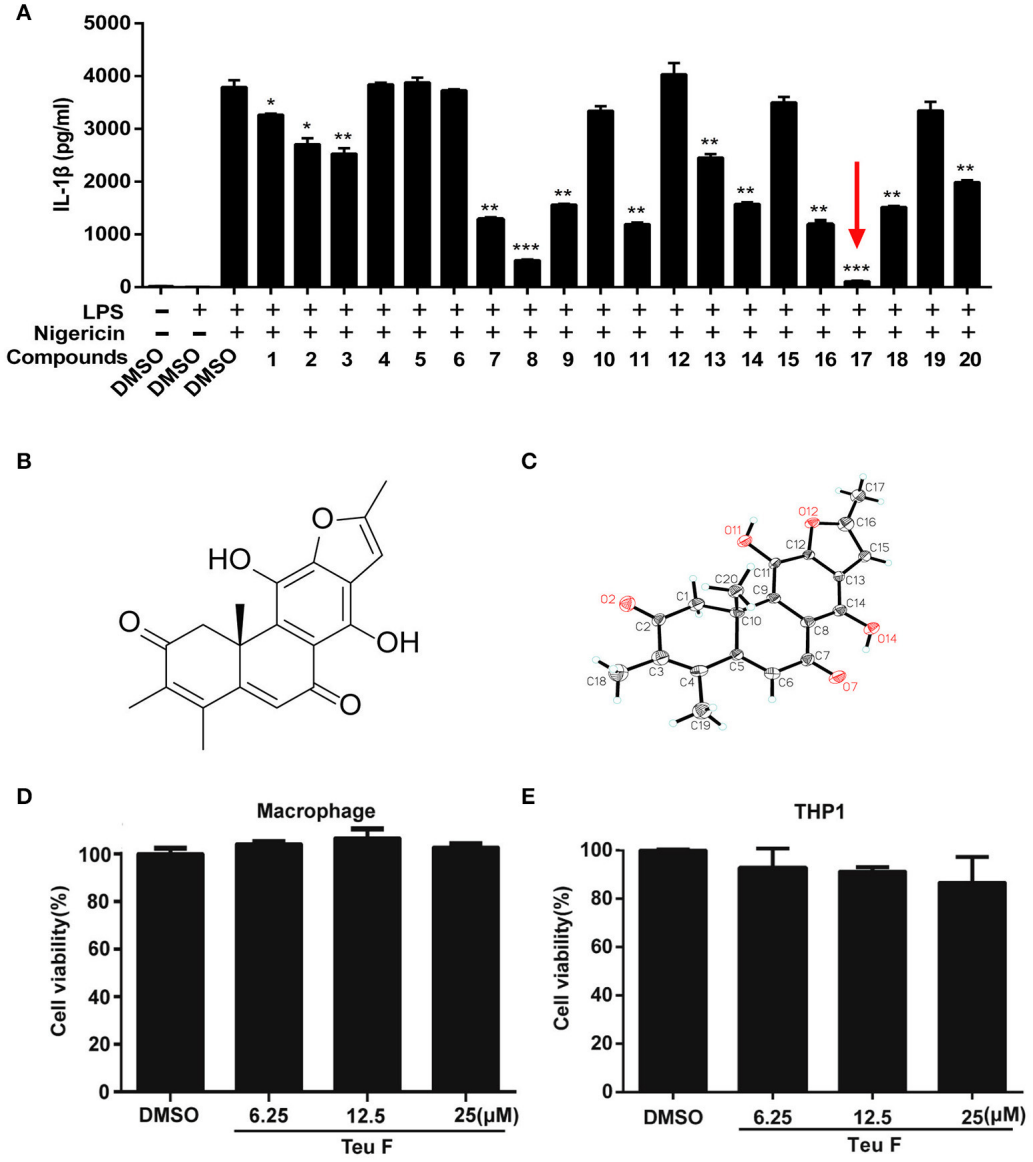
Screening potent inflammation inhibitor in natural compounds from *Premna szemaoensis Pei* extracts. **(A)** Mouse peritoneal macrophages were pretreated with DMSO or various compounds (25 μM) for 2 h, following stimulated with LPS (300 ng/ml) for 3 h and then treated with nigericin (10 μM) for 45 min. ELISA was used to analyze IL-1β secretion. **(B)** The chemical structure of Teuvincenone F. **(C)** The X-ray of Teuvincenone F. **(D,E)** Mouse peritoneal macrophages **(D)** and THP1 cells **(E)** were cultured with different concentrations of Teuvincenone F (0, 6.25, 12.25, 25 μM) for 12 h, the cell viability was determined by MTT assay. Data are shown as mean ± *SD* of one representative experiment in **(A,D–E)**. ^*^p < 0.05, ^**^p < 0.01, ^***^p < 0.001.

Inflammation regulation has a crucial effect on the process of many diseases, and the inflammatory cytokine IL-1β plays an important role in it. We isolated and purified a series of natural compounds (Table S2 and Figure S1), including diterpenoids and phenolic compounds, from the stems and leaves of *P. szemaoensis Pei*, and screened the highly effective anti-inflammatory compound. Previous finding demonstrated that nigericin is an essential activator to induce NLRP3 inflammasome activation (Mariathasan et al., 2006). So, we screened the natural compounds to assess the anti-inflammatory effect on LPS–induced IL-1β production after nigericin treatment. We found that Teuvincenone F (17#) could most significantly inhibit LPS–induced IL-1β production after nigericin stimulation (Figure 1A). Teuvincenone F is a diterpenoid compound with molecular formula of C_20_H_18_O_5_ and molecular weight of 338. Its chemical structure (Figure 1B) was deduced by extensive spectroscopic means, which was further determined by single-crystal X-ray crystallography and its crystallographic data have been deposited in the Cambridge Crystallographic Data Centre with deposition number CCDC 1547843 (Figure 1C and Figure S2). The Teuvincenone F potential cytotoxicity was evaluated via the MTT assay, incubating mouse primary macrophages (Figure 1D) and THP-1 cells (Figure 1E) for 12 h with or without Teuvincenone F. MTT assay demonstrated that cell viabilities both in macrophages and THP-1 cells were not obviously affected by the Teuvincenone F at different concentrations (0, 6.25, 12.5, 25 μM). Thus, the effects of Teuvincenone F (0–25 μM) on mouse primary macrophages and THP-1 cells were not attributable to cytotoxic effects. These results indicated that Teuvincenone F is an efficacious and potent inhibitor on LPS-induced inflammation.

### Teuvincenone F inhibits LPS-induced pro-inflammatory cytokine production in mouse peritoneal macrophages

**Figure 2:**
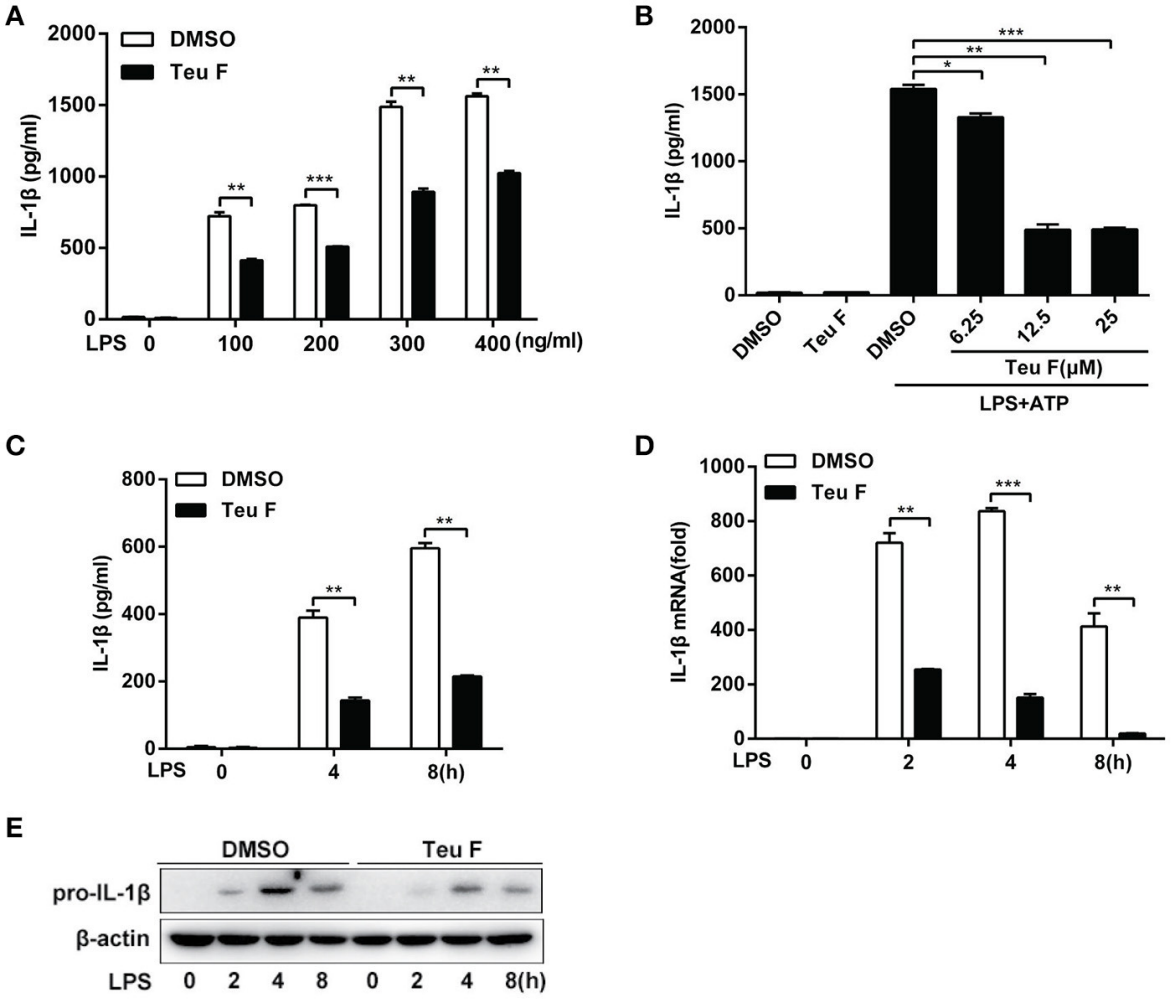
Teuvincenone F is an efficient inhibitor on LPS-induced IL-1β production in mouse peritoneal macrophages. **(A)** Mouse peritoneal macrophages were pretreated with DMSO or Teuvincenone F (25 μM) for 2 h, following stimulated with different concentrations of LPS (0, 100, 200, 300, 400 ng/ml) for 8 h and then treated with ATP (1 mM) for 45 min. ELISA was used to analyze IL-1β production. **(B)** Mouse peritoneal macrophages were pretreated with DMSO or different concentrations of Teuvincenone F (0, 6.25, 12.5, 25 μM) for 2 h, following stimulated with LPS (100 ng/ml) for 8 h and then treated with ATP (1 mM) for 45 min. IL-1β production was detected by ELISA. **(C)** Mouse peritoneal macrophages were pretreated with DMSO or Teuvincenone F (25 μM) for 2 h, following stimulated with LPS (100 ng/ml) for indicated hours and then treated with ATP (1 mM) for 45 min. ELISA was used to analyze IL-1β production. **(D, E)** Mouse peritoneal macrophages were pretreated with DMSO or Teuvincenone F (25 μM) for 2 h, following stimulated with LPS (100 ng/ml) for indicated hours, Q-PCR was used to analyze IL-1β mRNA expression **(D)** and immunoblot assay was used to analyze pro-IL-1β expression **(E)**. Data are shown as mean ± *SD* of one representative experiment in **(A–D)**. Similar results were obtained from three independent experiments **(E)**. ^*^p < 0.05, ^**^p < 0.01, ^***^p < 0.001.

To further study the role of Teuvincenone F in LPS-induced inflammation, we used different doses of LPS to stimulate Teuvincenone F-pretreated mouse peritoneal macrophages. The results showed that Teuvincenone F could obviously inhibit various doses of LPS-induced pro-inflammatory cytokine IL-1β production (Figure 2A). We also found that Teuvincenone F inhibitedLPS-induced IL-1β (Figure 2B) production in a dose-dependent manner. Consistently, ELISA experiments indicated that pretreatment of the cells with Teuvincenone F inhibited IL-1β (Figure 2C) production with LPS stimulation for different hours. Next, to confirm that Teuvincenone F inhibits LPS-induced IL-1β production in mRNA expression or protein maturation process, mouse peritoneal macrophages were pretreated with DMSO or Teuvincenone F, followed by stimulation with LPS for indicated hours. Q-PCR analysis showed that IL-1β mRNA expression (Figure 2D) was significantly decreased in macrophages pretreated with Teuvincenone F than the control group. In addition, we used immunoblot assay to analyze pro-IL-1β protein level after LPS stimulation with or without Teuvincenone F pretreatment and we observed a similar result that Teuvincenone F could inhibit pro-IL-1β production (Figure 2E). These data reveal that Teuvincenone F negatively regulates LPS-induced pro-inflammatory cytokine IL-1β production both in mRNA expression and protein maturation process.

**Figure 3:**
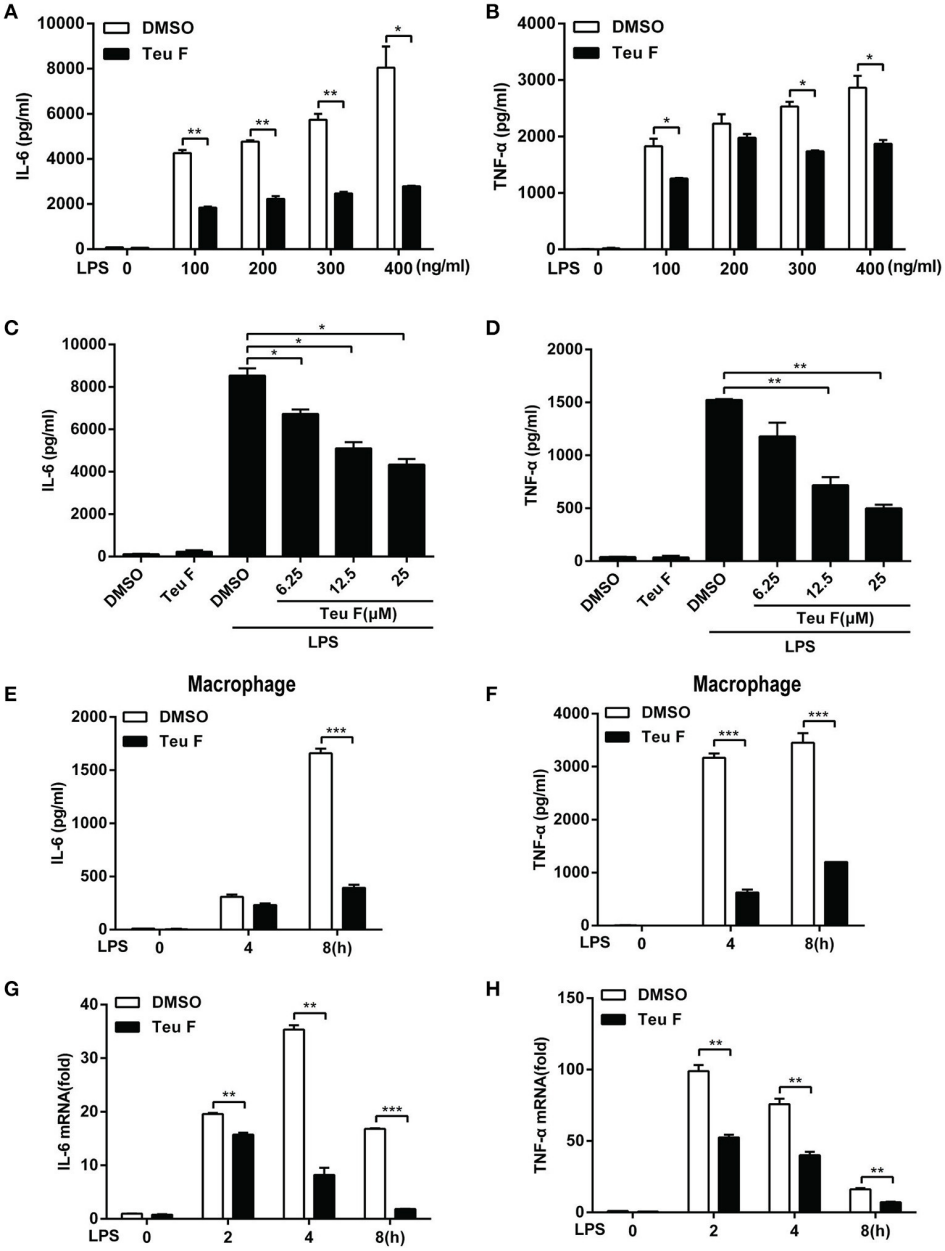
Teuvincenone F inhibits LPS-induced proinflammatory cytokine IL-6 and TNF-α production in mouse peritoneal macrophages. **(A,B)** Mouse peritoneal macrophages were pretreated with DMSO or Teuvincenone F (25 μM) for 2 h, following stimulated with different concentrations of LPS (0, 100, 200, 300, 400 ng/ml) for 8 h and ELISA was used to analyze IL-6 **(A)** and TNF-α **(B)** production. **(C,D)** Mouse peritoneal macrophages were pretreated with DMSO or different concentrations of Teuvincenone F (0, 6.25, 12.5, 25 μM) for 2 h, following stimulated with LPS (100 ng/ml) for 8 h and ELISA was used to analyze IL-6 **(C)** and TNF-α **(D)** production. **(E,F)** Mouse peritoneal macrophages were pretreated with DMSO or Teuvincenone F (25 μM) for 2 h, following stimulated with LPS (100 ng/ml) for indicated hours and ELISA was used to analyze IL-6 **(E)** and TNF-α **(F)** production. **(G,H)** Mouse peritoneal macrophages were pretreated with DMSO or Teuvincenone F (25 μM) for 2 h, following stimulated with LPS (100 ng/ml) for indicated hours, Q-PCR was used to analyze IL-6 **(G)** and TNF-α **(H)** mRNA expression. Data are shown as mean ± *SD* of one representative experiment in **(A–H)**. ^*^p < 0.05, ^**^p < 0.01, ^***^p < 0.001.

Mouse peritoneal macrophages were stimulated with different doses of LPS with Teuvincenone F pretreatment, and the results also showed that Teuvincenone F significantly inhibited various doses of LPS-induced pro-inflammatory cytokines IL-6 (Figure 3A) and TNF-α (Figure 3B) production. Again, Teuvincenone F inhibited LPS-induced pro-inflammatory cytokines IL-6 (Figure 3C) and TNF-α (Figure 3D) production in a dose-dependent manner. In addition, macrophages were stimulated with LPS for different hours, IL-6 (Figure 3E) and TNF-α (Figure 3F) production was dramatically decreased in macrophages pretreated with Teuvincenone F as compared with control group. Besides, we obtained the similar results for IL-6 (Figure 3G) and TNF-α (Figure 3H) mRNA level by Q-PCR analysis. Taken together, these data provide additional evidence that Teuvincenone F negatively regulates LPS-induced pro-inflammatory cytokine production.

### Teuvincenone F suppresses LPS-induced NF-κB activation by attenuating K63-linked ubiquitination of NEMO in mouse peritoneal macrophages

**Figure 4:**
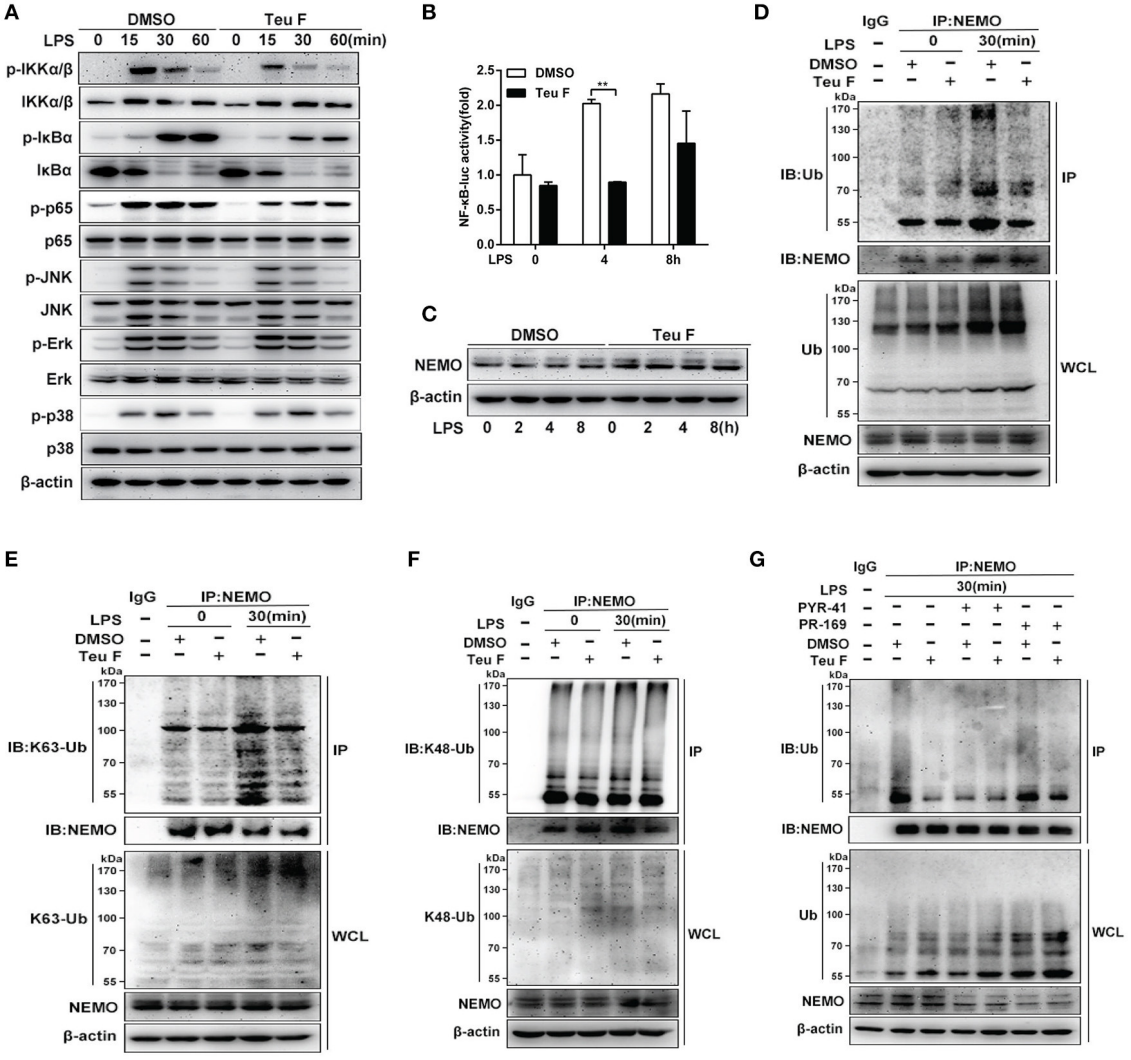
Teuvincenone F suppresses LPS-induced NF-κB activation by decreasing K63-linked ubiquitination of NEMO in mouse macrophages. **(A)** Mouse peritoneal macrophages were pretreated with DMSO or Teuvincenone F (25 μM) for 2 h, following stimulated with LPS (100 ng/ml) for indicated hours, immunoblot assay was used to analyze NF-κB and MAPKs signaling pathways. Phosphorylated IKKα/β and total form of IKKα/β, phosphorylated JNK and total form of JNK, phosphorylated p38 and total form of p38, came from the same immunoblot, phosphorylated IκBα and total form of IκBα, phosphorylated p65 and total form of p65, phosphorylated Erk and total form of Erk, came from the replicate immunoblot. **(B)** RAW 264.7 cells were transfected with the NF-κB luciferase reporter plasmid (90 ng), TK plasmid (10 ng). Twenty-four hours transfection, cells was pretreated with DMSO or Teuvincenone F (25 μM) for 2 h, following stimulated with LPS (100 ng/ml) for indicated hours, then detected NF-κB activity by luciferase assay. **(C)** Mouse peritoneal macrophages were pretreated with DMSO or Teuvincenone F (25 μM) for 2 h, following stimulated with LPS (100 ng/ml) for indicated hours, immunoblot assay was used to analyze NEMO expression. **(D–F)** Mouse peritoneal macrophages were pretreated with DMSO or Teuvincenone F (25 μM) for 2 h, following stimulated with LPS (100 ng/ml) for indicated hours, and then subjected to immunoprecipitation with anti-NEMO antibody followed by immunoblot analysis with specific anti-Ub **(D)**, anti-K63-Ub **(E)**, or anti-K48-Ub **(F)** antibodies. **(G)** Ubiquitination inhibitor PYR-41 and deubiquitination inhibitor PR-619 pretreated macrophages with or without Teuvincenone F (25 μM) for 2 h, following stimulated with LPS (100 ng/ml) for 30 min, and then subjected to immunoprecipitation with anti-NEMO antibody followed by immunoblot analysis with specific antibodies. Similar results were obtained from three independent experiments **(A,C–G)**. Data are shown as mean ± *SD* of one representative experiment in **(B)**. ^**^p < 0.01.

Previous study showed that NF-κB activation was important for LPS-induced inflammatory cytokines production (Mitchell et al., 2016). To verify involved signaling pathway, mouse peritoneal macrophages were pretreated with DMSO or Teuvincenone F, followed by stimulation with LPS, immunoblot assay was used to analyze NF-κB and MAPKs signaling pathways. The results showed that Teuvincenone F significantly suppressed NF-κB signaling pathway activation, including decreasing phosphorylation of IKKα/β, IκBα, and p65, but not the MAPKs signaling pathways in mouse peritoneal macrophages (Figure 4A). The luciferase activity assay was used to analyze NF-κB activation in RAW 264.7 cells, and the result also showed that pretreatment of the cells with Teuvincenone F inhibited NF-κB activation after LPS stimulation (Figure 4B).

Since Teuvincenone F inhibited the phosphorylation of IKKα/β, IκBα, and p65, and previous study showed that ubiquitination of NEMO was indispensable for phosphorylation IKKα/β and NF-κB activation (Temmerman et al., 2006; Wu et al., 2006), we assumed that Teuvincenone F might affect NEMO activation. To investigate the underlying mechanisms involved in these processes, we used immunoblot to analyze the NEMO protein level after LPS stimulation with or without pretreatment with Teuvincenone F and the results showed that Teuvincenone F didn't affect NEMO protein expression in mouse peritoneal macrophages (Figure 4C). However, our study indicated that polyubiquitination of NEMO was decreased after LPS stimulation with Teuvincenone F pretreatment in mouse peritoneal macrophages (Figure 4D). Furthermore, to investigate the form of ubiquitination chains linked to NEMO, we used the K48 and K63 sites ubiquitin specific antibody to detect the ubiquitination of NEMO after LPS stimulation with Teuvincenone F pretreatment. Co-immunoprecipitation experiments showed that Teuvincenone F decreased K63-linked (Figure 4E) but not K48-linked (Figure 4F) polyubiquitination of NEMO. Next, to further investigated that whether Teuvincenone F regulated the ubiquitination or deubiquitination of NEMO in this process. We used the ubiquitination inhibitor PYR-41 or deubiquitination inhibitor PR-619 to pretreat macrophages with or without Teuvincenone F. We found that PR-619 could not affect the NEMO ubiquitination decreased caused by Teuvincenone F (Figure 4G), the result suggesting that Teuvincenone F might regulate the process of NEMO ubiquitination, not the process of NEMO deubiquitination. Taken together, these findings reveal that Teuvincenone F suppresses LPS-induced NF-κB activation by attenuating K63-linked polyubiquitination of NEMO in mouse peritoneal macrophages.

### Teuvincenone F inhibits LPS-induced pro-inflammatory cytokine production by suppressing NF-κB activation via attenuating ubiquitination of NEMO in THP-1 cells

**Figure 5:**
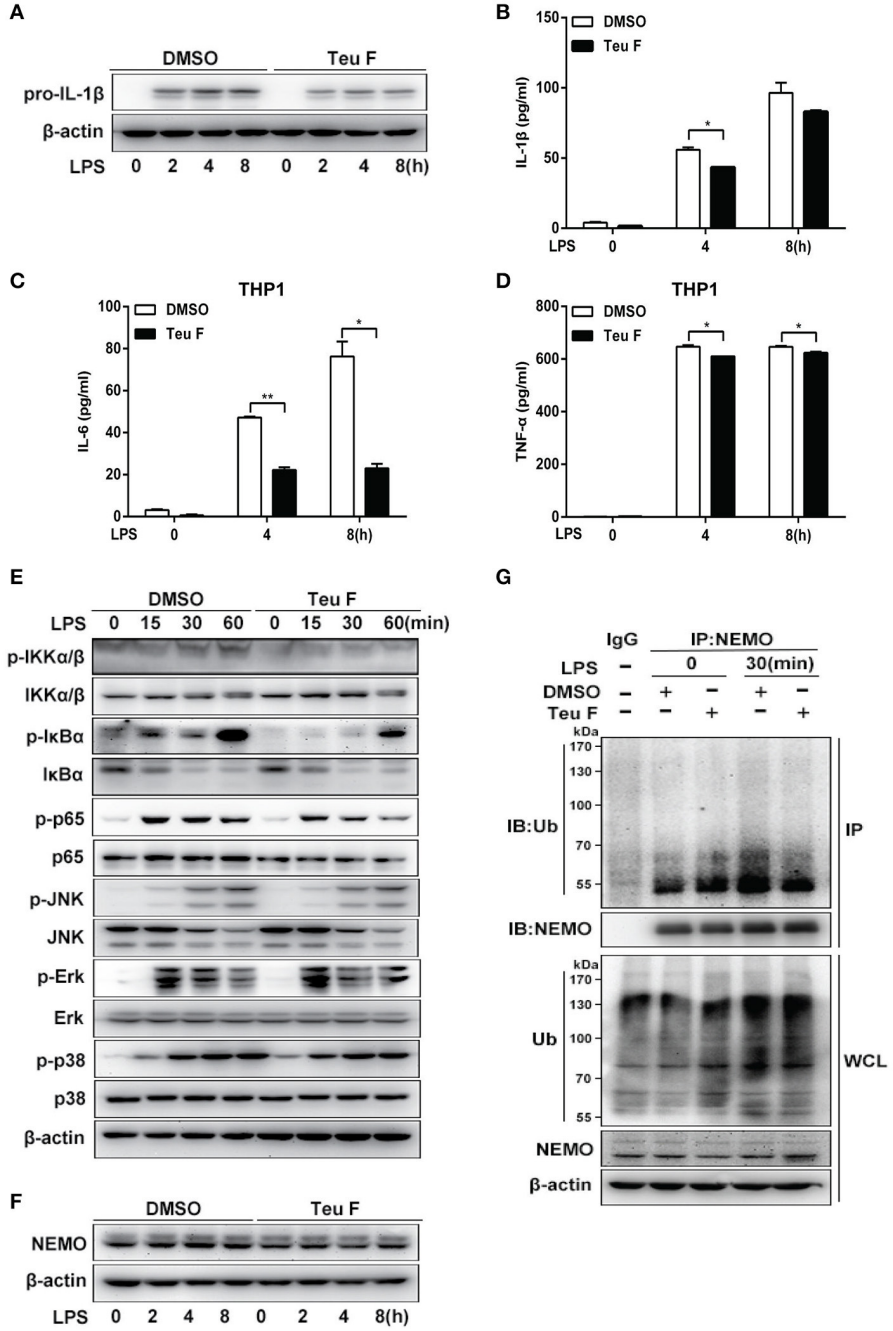
Teuvincenone F inhibits LPS-induced proinflammatory cytokine production by suppressing NF-κB activation via decreasing ubiquitination of NEMO in THP1 cells. **(A)** THP1 cells were pretreated with DMSO or Teuvincenone F (25 μM) for 2 h, following stimulated with LPS (100 ng/ml) for indicated hours, immunoblot assay was used to analyze pro-IL-1β expression. **(B)** THP1 cells were pretreated with DMSO or Teuvincenone F (25 μM) for 2 h, following stimulated with LPS (100 ng/ml) for indicated hours and then treated with ATP (1 mM) for 45 min. ELISA was used to analyze IL-1β production. **(C,D)** THP1 cells were pretreated with DMSO or Teuvincenone F (25 μM) for 2 h, following stimulated with LPS (100 ng/ml) for indicated hours and ELISA was used to analyze IL-6 **(C)** and TNF-α **(D)** production. **(E)** THP1 cells were pretreated with DMSO or Teuvincenone F (25 μM) for 2 h, following stimulated with LPS (100 ng/ml) for indicated hours, immunoblot assay was used to analyze NF-κB and MAPKs signaling pathways. Phosphorylated p65 and total form of p65, phosphorylated JNK and total form of JNK, phosphorylated Erk and total form of Erk, phosphorylated p38 and total form of p38, came from the replicate immunoblot, phosphorylated IKKα/β and total form of IKKα/β, phosphorylated IκBα and total form of IκBα, came from the replicate immunoblot. **(F)** THP1 cells were pretreated with DMSO or Teuvincenone F (25 μM) for 2 h, following stimulated with LPS (100 ng/ml) for indicated hours, immunoblot assay was used to analyze NEMO. (G) THP1 cells were pretreated with DMSO or Teuvincenone F (25 μM) for 2 h, following stimulated with LPS (100 ng/ml) for indicated hours, and then subjected to immunoprecipitation with anti-NEMO antibody followed by western blot analysis with anti-Ub antibody. Similar results were obtained from three independent experiments **(A,E–G)**. Data are shown as mean ± *SD* of one representative experiment in **(B–D)**. ^*^p < 0.05, ^**^p < 0.01.

Our studies showed that Teuvincenone F negatively regulates LPS-induced pro-inflammatory cytokines production in mouse peritoneal macrophages. As a potential natural compound used to treat human diseases caused by inflammation, we used the human monocytic cell line THP-1 to do the next experiments. As expected, Q-PCR analysis showed that LPS-induced IL-1β mRNA expression (Figure S3A) was significantly decreased in THP-1 cells pretreated with Teuvincenone F compared with the control group. Next, we also found that Teuvincenone F dramatically suppressed inflammatory cytokines IL-6 (Figure S3B) and TNF-α (Figure S3C) expression in LPS-treated THP-1 cells. We observed similar results by immunoblot analysis of pro-IL-1β protein level after LPS stimulation with Teuvincenone F pretreatment (Figure 5A). ELISA experiments also confirmed these results. Pretreatment of Teuvincenone F inhibited LPS-induced IL-1β (Figure 5B), IL-6 (Figure 5C), and TNF-α (Figure 5D) production in THP-1 cells. Similarly, we found that Teuvincenone F significantly suppressed NF-κB activation, including decreased phosphorylations of IKKα/β, IκBα, and p65, but not the MAPKs signaling pathways in THP-1 cells (Figure 5E). In addition, our study also found that Teuvincenone F didn't affect NEMO protein stabilization (Figure 5F), but decreased the ubiquitination of NEMO in LPS-treated THP-1 cells (Figure 5G). These results indicate that the Teuvincenone F negatively regulates LPS-induced inflammatory cytokines production by suppressing NF-κB activation via attenuating ubiquitination of NEMO in THP-1 cells.

### Teuvincenone F suppresses LPS-induced NLRP3 inflammasome activation by inhibiting NLRP3 expression

**Figure 6:**
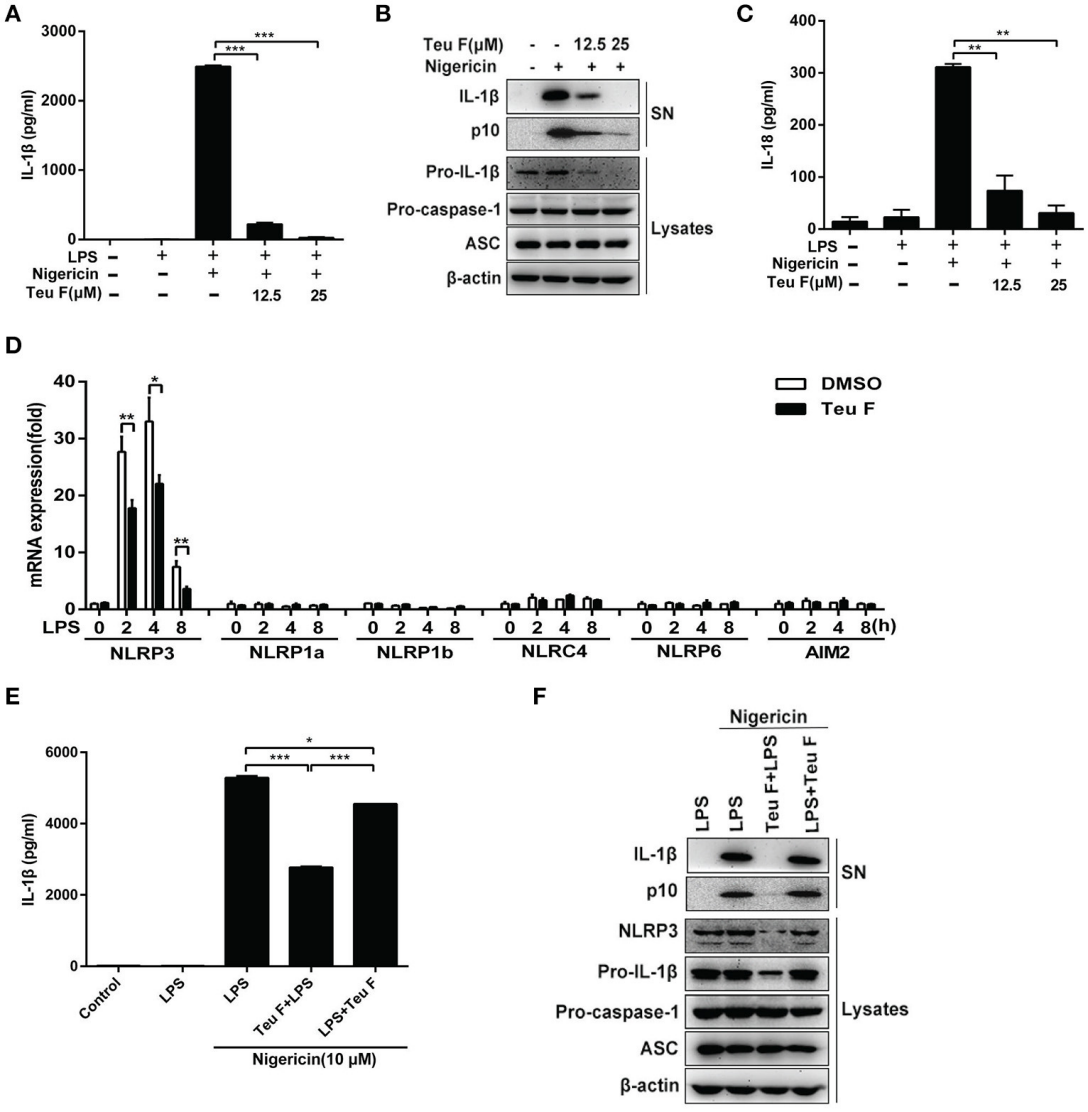
Teuvincenone F suppresses LPS-induced NLRP3 inflammasome activation by inhibiting NLRP3 expression. **(A)** Mouse peritoneal macrophages were pretreated with various doses of Teuvincenone F (0, 12.5, 25 μM) for 2 h, following stimulated with LPS (300 ng/ml) for 3 h and then treated with nigericin (10 μM) for 45 min. ELISA was used to analyze IL-1β secretion. **(B)** Mouse peritoneal macrophages were pretreated with various doses (0, 12.5, 25 μM) of Teuvincenone F for 2 h, following stimulated with LPS (300 ng/ml) for 3 h and then treated with nigericin (10 μM) for 45 min. Immunoblot analyze IL-1β and cleaved caspase-1 (p10) in culture supernatants (SN) and the precursors of IL-1β (pro-IL-1β) and caspase-1 (pro-caspase-1) in lysates of these cells (Lysates). **(C)** Mouse peritoneal macrophages were pretreated with various doses of Teuvincenone F (0, 12.5, 25 μM) for 2 h, following stimulated with LPS (300 ng/ml) for 3 h and then treated with nigericin (10 μM) for 45 min. ELISA was used to analyze IL-18 secretion. **(D)** Mouse peritoneal macrophages were pretreated with DMSO or Teuvincenone F (25 μM) for 2 h, following stimulated with LPS (100 ng/ml) for indicated hours, Q-PCR was used to analyze NLRP3, NLRP1a, NLRP1b, NLRC4, NLRP6, and AIM2 mRNA expression. **(E)** Mouse peritoneal macrophages were pretreated with Teuvincenone F (25 μM) for 2 h, following stimulated with LPS (300 ng/ml) for 3 h or LPS-primed mouse peritoneal macrophages (3 h) treated with Teuvincenone F (25 μM), and then stimulated with nigericin (10 μM) for 45 min. ELISA was used to analyze IL-1β secretion. **(F)** Mouse peritoneal macrophages were pretreated with Teuvincenone F (25 μM) for 2 h, following stimulated with LPS (300 ng/ml) for 3 h or LPS-primed mouse peritoneal macrophages (3 h) treated with Teuvincenone F (25 μM), and then stimulated with nigericin (10 μM) for 45 min. Immunoblot analyze IL-1β and cleaved caspase-1 (p10) in culture supernatants (SN) and the precursors of IL-1β (pro-IL-1β) and caspase-1 (pro-caspase-1) in lysates of these cells (Lysates). Data are shown as mean ± *SD* of one representative experiment in **(A,C–E)**. Similar results were obtained from three independent experiments **(B,F)**. ^*^p < 0.05, ^**^p < 0.01, ^***^p < 0.001.

Nigericin is an activator of NLRP3 inflammasome and our studies showed that Teuvincenone F could inhibit LPS–induced IL-1β production after nigericin stimulation. So, Teuvincenone F might affect NLRP3 inflammasome activation to regulate IL-1β maturation and secretion. To further investigate the effect of Teuvincenone F on NLRP3 inflammasome activation and IL-1β maturation secretion. Firstly, we examined whether Teuvincenone F could inhibit caspase-1 and IL-1β maturation. The results showed that pretreatment with Teuvincenone F and then LPS simulation blocked caspase-1 cleavage and IL-1β production in a dose-dependent manner in mouse peritoneal macrophages (Figures 6A,B). We also found that Teuvincenone F inhibited pro-IL-1β production (Figure 6B). Similarly, Teuvincenone F also inhibited nigericin-induced IL-18 secretion, another important inflammasome-dependent cytokine (Figure 6C), but we didn't find obvious difference at IL-18 mRNA level (data not shown). Previous study showed that cell priming through multiple signaling receptors promoted NF-κB activation to induce NLRP3 expression, which is a critical checkpoint for NLRP3 activation. Macrophages need to acquire a licensing signal provided by a transcriptionally active signaling receptor that enables them to respond to NLRP3 activators (Bauernfeind et al., 2009). In our study, we assumed that Teuvincenone F could suppress LPS-induced NF-κB signaling pathway and inhibit NLRP3 inflammasome activation. To verify this hypothesis, mouse peritoneal macrophages were pretreated with DMSO or Teuvincenone F, followed by stimulation with LPS, Q-PCR assay was used to analyze NLRP3 mRNA expression. The results showed that Teuvincenone F significantly suppressed NLRP3 mRNA expression in mouse peritoneal macrophages, without affecting NLRP1a, NLRP1b, NLRC4, NLRP6, and AIM2 mRNA expression (Figure 6D). These data are consistent with previous study (Bauernfeind et al., 2011). To ascertain that Teuvincenone F inhibits NLRP3 inflammasome by a direct way to inhibit NLRP3 inflammasome activation or by an indirect way to inhibit NLRP3 mRNA expression, we performed the rescue experiment and found that Teuvincenone F treatment on the LPS-primed mouse peritoneal macrophages significantly increased IL-1β secretion and caspase-1 activation (Figures 6E,F) accompanied with decreased NLRP3 protein expression (Figure 6F). In summary, these data suggest that Teuvincenone F suppresses LPS-induced NLRP3 inflammasome activation by inhibiting NLRP3 expression.

### Teuvincenone F relieves LPS-induced inflammation *In vivo*

**Figure 7:**
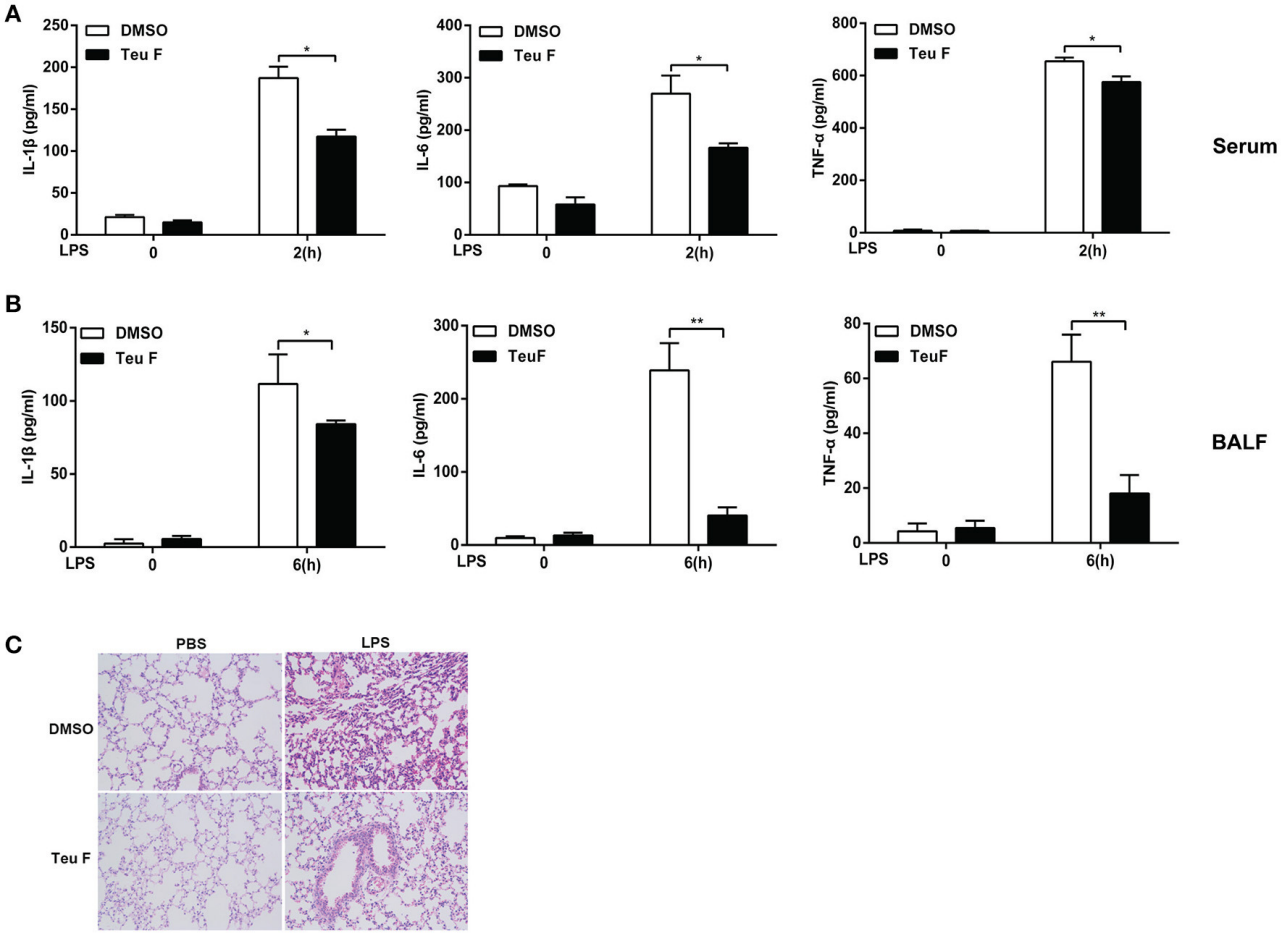
Teuvincenone F relieves LPS-induced inflammation in mice. **(A)** C57BL/6 mice (*n* = 3–5 mice/group) were intraperitoneal injected with DMSO or Teuvincenone F (5 mg/kg, intraperitoneal injection) for 30 min, and challenged with PBS or LPS (10 mg/kg, intraperitoneal injection). Two hours later, mice were sacrificed and ELISA was used to analyze IL-1β, IL-6, and TNF-α cytokine concentration in the peripheral blood. **(B,C)** C57BL/6 mice (*n* = 3–5 mice/group) were intraperitoneal injected with DMSO or Teuvincenone F (5 mg/kg, intraperitoneal injection) for 30 min, and challenged with PBS or LPS (20 mg/kg, intraperitoneal injection). Six hours later, mice were sacrificed and collected the bronchoalveolar lavage fluid (BALF), ELISA was used to analyze IL-1β, IL-6, and TNF-α cytokine concentration **(B)**, H&E staining of lung sections (200×) **(C)**. Data are shown as mean ± *SD* of one representative experiment in **(A,B)**. ^*^p < 0.05, ^**^p < 0.01.

Sepsis is a systemic inflammatory response syndrome that often results from the infection of pathogen. It is characterized by overproduction of inflammatory cytokines and leads to the lethal multiple organ damage after pathogen components stimulation. So, we next assessed the *in vivo* function of Teuvincenone F using a murine model of inflammation induced by LPS. We found that mice pretreated with Teuvincenone F had a decreased production of IL-1β, IL-6, and TNF-a (Figure 7A) in the peripheral blood after injected intraperitoneally with LPS. In addition, compared with the control group, the mice pretreated with Teuvincenone F produced less pro-inflammatory cytokine IL-1β, IL-6, and TNF-a (Figure 7B) in the bronchoalveolar lavage fluid and exhibited less inflammatory cells infiltration in lung (Figure 7C). These data indicate that Teuvincenone F protects the mice more resistant to LPS induced inflammation and lung injury *in vivo*.

## Discussion

Productive inflammation is important not only for the body to fight infection and damage, but also to avoid inflammatory disease and this process is regulated in a complex and distinct manner. However, uncontrolled and excessive immune response can cause pathological immunity or autoimmune diseases to the host (Bach, 2005). So it's important to ensure proper production of inflammatory cytokines following pathogen infection. As the NF-κB pathway represents one of the key inflammatory signaling pathways in the body, it is not surprising that there are many regulatory mechanisms to balance the host inflammatory response, including Cdc37 and Hsp90, which is important for TNFα-induced recruitment and IKKs complex activation, then resulting in NF-κB activation (Chen et al., 2002), Casein kinase 1α could also regulate activation of NF-κB signaling induced by antigen-receptor and the survival of human lymphoma cell (Bidere et al., 2009). In this study, we identified, for the first time, that Teuvincenone F is a highly effective natural compound to suppress LPS-induced inflammation and NLRP3 inflammasome activation via attenuating K63-linked ubiquitination of NEMO.

Inflammation causes many diseases, such as sepsis, which seriously threats to human health. So, the anti-inflammatory drug research of related diseases is urgently needed. Previous studies showed that ingredients extracted from plants had many pharmacological functions. Diterpenoid compounds, as an important part of ingredients extracted from plants, had received many studies for anti-tumor, anti-viral, and anti-inflammation effects. Diterpenoid purified from *Isodon melissoides* can induce redox imbalance to promote leukemic cell apoptosis and exhibit synergy with other anti-cancer drugs (Yu et al., 2007). Diterpenoid compound 8,10,18-trihydroxy-2,6-dolabelladiene not only inhibits HIV-1 reverse transcription, but also blocks HIV-1 replication at a post transcriptional step (Cirne-Santos et al., 2006). 15,16-epoxy-3α-hydroxylabda-8,13(16),14-trien-7-one, as an anti-inflammatory agent, could significantly suppress LPS-induced crucial proinflammatory mediators, COX-2, iNOS, and TNF-α, by inhibiting of the MAPK, NF-κB, and Akt signaling pathways (Khan et al., 2012). In the current study, we isolated and purified a series of compounds from *P. szemaoensis Pei* and found that the diterpenoid component, Teuvincenone F, could significantly inhibit LPS–induced IL-1β production. These results suggest that Teuvincenone F has an ideal effect on anti-inflammation.

We investigated and defined the possible mechanism of Teuvincenone F on LPS-induced inflammation. We first confirmed that Teuvincenone F inhibited LPS-induced phosphorylations of IKKα/β, IκBα, and p65, suppressing inflammatory cytokines production, including IL-1β, IL-6, and TNF-α, with no effect on the MAPKs pathways. Previous study showed that ubiquitination of NEMO was very important for NF-κB activation (Wu et al., 2006). Our ubiquitination assay showed that Teuvincenone F obvious attenuated polyubiquitination of NEMO, especially K63-linked polyubiquitination of NEMO after LPS stimulation, without affecting total NEMO protein level. Collectively, our results indicate that Teuvincenone F attenuates K63-linked polyubiquitination of NEMO to suppress LPS-induced NF-κB signaling pathway activation. What's more, many studies have revealed that a strong role of liner polyubiquitination of NEMO in the inflammatory response, and demonstrated this process is indispensable for NF-κB signaling pathway activation (Sebban et al., 2006; Jun et al., 2013). Studies showed that LUBAC (linear ubiquitin chain assembly complex) was important for the linear polyubiquitylation of NEMO, these results implied that HOIL-1L and HOIP might play the E3 ligase role in NEMO liner polyubiquitination process (Tokunaga et al., 2009). The Teuvincenone F might also affect the liner polyubiquitination of NEMO after LPS stimulation and it is in need of further confirmation. Besides, USP18 could inhibit K63-linked polyubiquitination of NEMO, and then negatively regulating the NF-κB signaling pathways (Yang et al., 2015). It is known that inflammasomes are important for inflammatory cytokines IL-1β/IL-18 maturation and secretion (Schroder and Tschopp, 2010). We found that Teuvincenone F could inhibit NF-κB activation, and previous study showed that LPS promoted NF-κB activation to induce NLRP3 expression, which is a critical checkpoint for NLRP3 activation (Bauernfeind et al., 2009). However, NLRP3 is unique among the known inflammasome pathways in its specific requirement of a proinflammatory priming signal (Bauernfeind et al., 2011). So, Teuvincenone F might inhibit NLRP3 inflammasome activation by suppressing NLRP3 mRNA expression, and then decreasing IL-1β/IL-18 maturation and secretion. Indeed, we demonstrated that Teuvincenone F could inhibit NLRP3 mRNA expression, but didn't affect NLRP1a, NLRP1b, NLRC4, NLRP6, and AIM2 mRNA expression after LPS stimulation by Q-PCR assay. In addition, immunoblot and ELISA assays also confirmed that Teuvincenone F decreased NLRP3 expression, resulted in less NLRP3 inflammasome activation, followed by suppressed IL-1β/IL-18 maturation and secretion.

**Figure 8:**
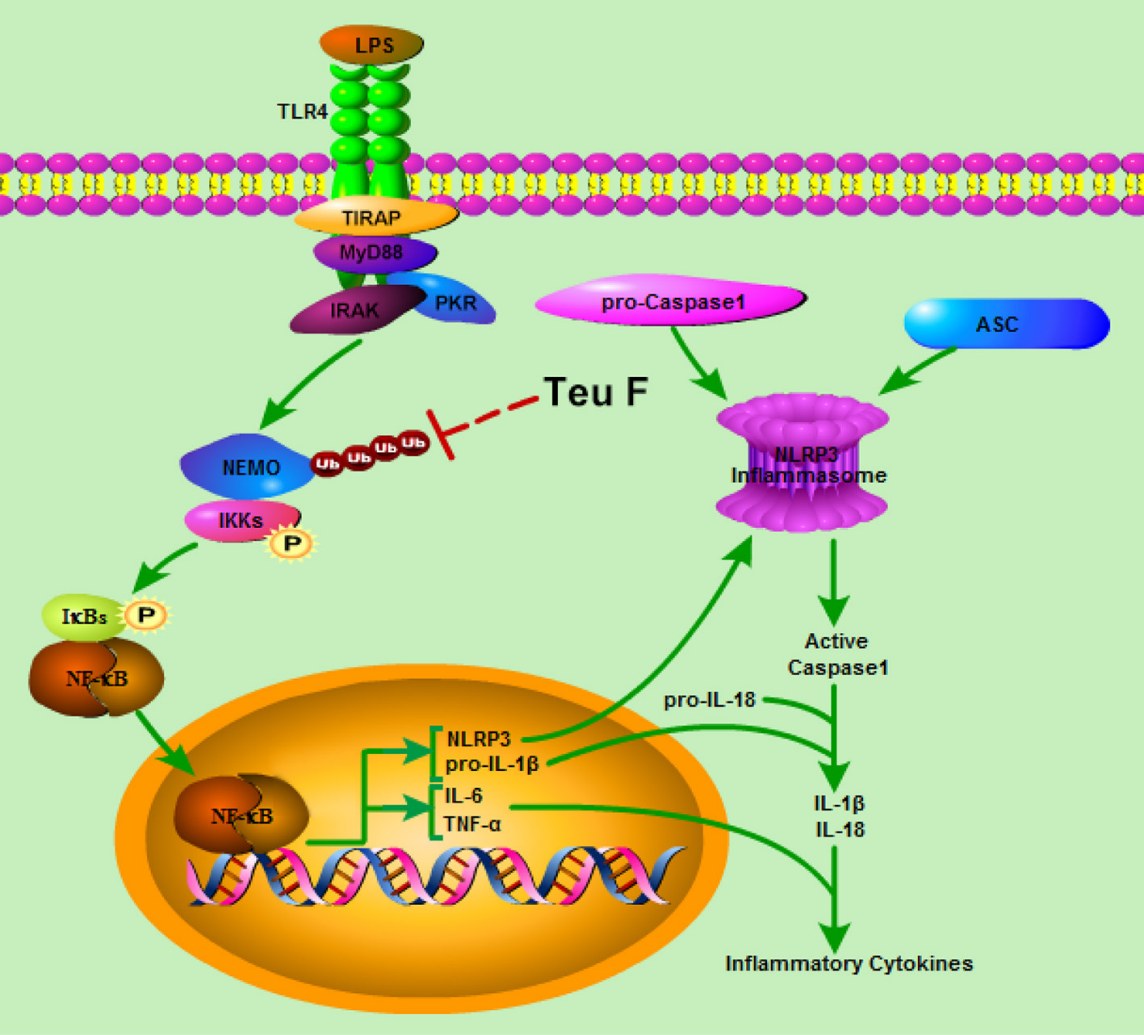
Model of Teuvincenone F suppresses LPS-induced inflammatory cytokine production and NLRP3 inflammasome activation via attenuating K63-linked ubiquitination of NEMO. Upon LPS stimulated, TLR4 is activated, and then activates its downstream signaling pathways, promotes NEMO K63-linked ubiquitination, IKKα/β and NF-κB phosphorylation, following promotes LPS-induced inflammatory cytokine production and NLRP3 expression. Teuvincenone F suppresses LPS-induced K63-linked ubiquitination of NEMO and then inhibits IL-1β, IL-6, TNF-α, and NLRP3 mRNA expression, prevented inflammatory cytokines IL-6 and TNF-α production. Besides, decreasing NLRP3 expression affected NLRP3 inflammasome assemble and resulted in inhibiting NLRP3 inflammasome activation, following suppresses IL-1β/IL-18 maturation. In summary, Teuvincenone F suppresses LPS-induced inflammatory cytokines production via attenuating K63-linked ubiquitination of NEMO.

In conclusion, we have demonstrated that Teuvincenone F suppresses LPS-induced inflammation and NLRP3 inflammasome activation through blocking NF-κB signaling pathway in macrophages. This process depends on Teuvincenone F attenuation of NEMO K63-linked polyubiquitination, as illustrated in Figure 8. This working model explains how mice pretreated with Teuvincenone F show more resistance to LPS challenge. These findings provide new pharmacological insight into the negative regulation of NF-κB-mediated inflammation in innate immune responses, highlighting Teuvincenone F as a potential new anti-inflammatory drug for the treatment of infection-related inflammatory and autoimmune diseases.

## Author contributions

WC, WX, and XibZ participated in the design of the study. XibZ, DP, ZZ, HZ, and YS performed research. XibZ, HL, XibZ, RZ, and WC analyzed data. XibZ, JS, WX, and WC wrote the paper.

### Conflict of interest statement

The authors declare that the research was conducted in the absence of any commercial or financial relationships that could be construed as a potential conflict of interest.
