# Supplementary material for: Teuvincenone F Suppresses LPS-Induced Inflammation and NLRP3 Inflammasome Activation by Attenuating NEMO Ubiquitination
Source: Front Pharmacol. 2017 Aug 23;8:565. doi: 10.3389/fphar.2017.00565 (PMC5572209; doi:10.3389/fphar.2017.00565)
Supplement: Supplementary file 1 [file Image1.PDF]

Supplementary material

Figure S1

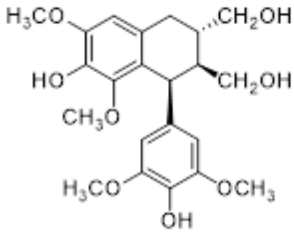

1

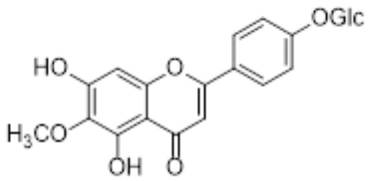

2

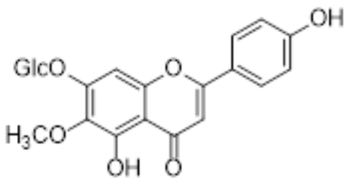

3

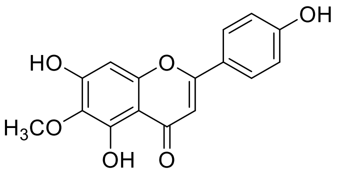

4

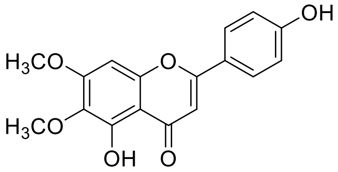

5

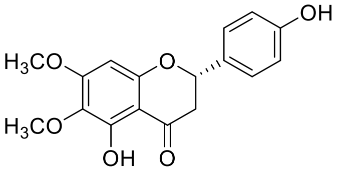

6

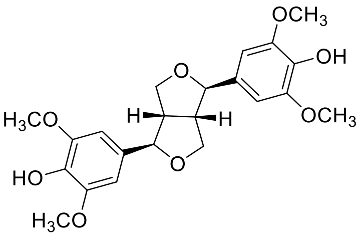

7

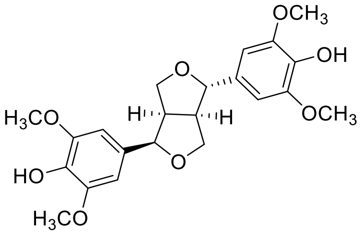

8

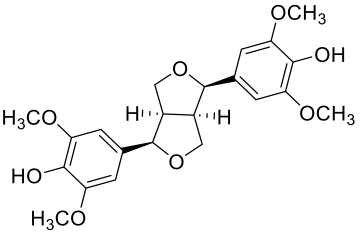

9

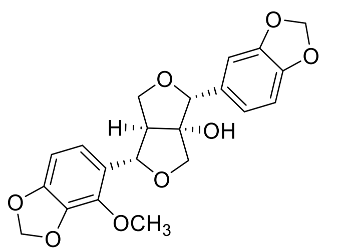

10

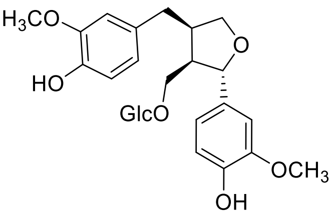

11

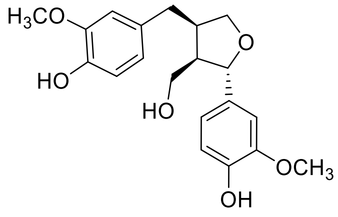

12

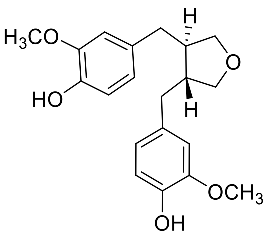

13

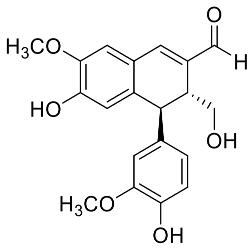

14

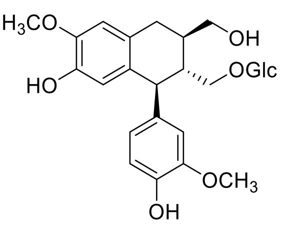

15

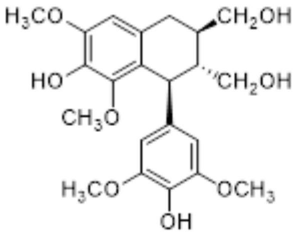

16

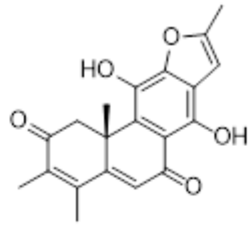

17

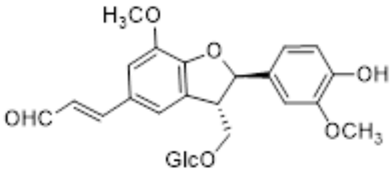

18

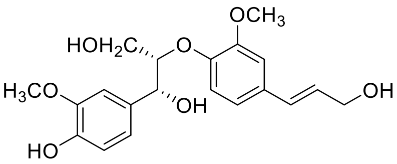

19

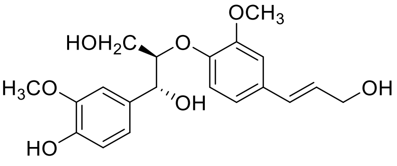

20

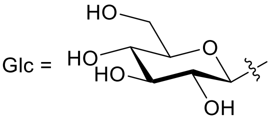

Supplementary Figure S1. Structures of 20 compounds from *P. szemaoensis*.
