# Supplementary material for: Teuvincenone F Suppresses LPS-Induced Inflammation and NLRP3 Inflammasome Activation by Attenuating NEMO Ubiquitination
Source: Front Pharmacol. 2017 Aug 23;8:565. doi: 10.3389/fphar.2017.00565 (PMC5572209; doi:10.3389/fphar.2017.00565)
Supplement: Supplementary file 2 [file Image2.PDF]

Supplementary material

Figure S2

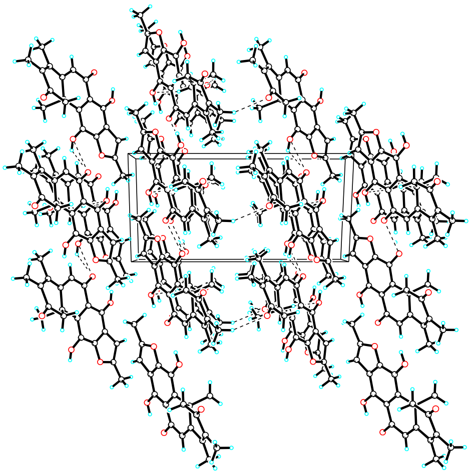

Supplementary Figure S2. X-ray structure and crystal data of Teuvincenone F.

Crystal data for Teuvincenone F: $C_{20}H_{18}O_5$, M = 338.34, triclinic, a = 7.255(2) Å, b = 8.226(2) Å, c = 15.241(6) Å, α = 90.02(3)°, β = 90.82(3)°, γ = 115.95(2)°, V = 817.7(5) Å^3, T = 100(2) K, space group $P1$, Z = 2, μ(CuKα) = 0.814 mm^{-1}, 4371 reflections measured, 2976 independent reflections (R_{int} = 0.0686). The final R_1 values were 0.1987 ($I > 2\sigma(I)$). The final $wR(F^2)$ values were 0.4522 ($I > 2\sigma(I)$). The final R_1 values were 0.2288 (all data). The final $wR(F^2)$ values were 0.4790 (all data). The goodness of fit on F^2 was 1.964. Flack parameter = -0.1(10). Crystallographic data for the structure of teuvincenone F have been deposited in the Cambridge Crystallographic Data Centre (deposition number: CCDC 1547843).
