# Supplementary material for: Teuvincenone F Suppresses LPS-Induced Inflammation and NLRP3 Inflammasome Activation by Attenuating NEMO Ubiquitination
Source: Front Pharmacol. 2017 Aug 23;8:565. doi: 10.3389/fphar.2017.00565 (PMC5572209; doi:10.3389/fphar.2017.00565)
Supplement: Supplementary file 3 [file Image3.PDF]

Supplementary material

Figure S3

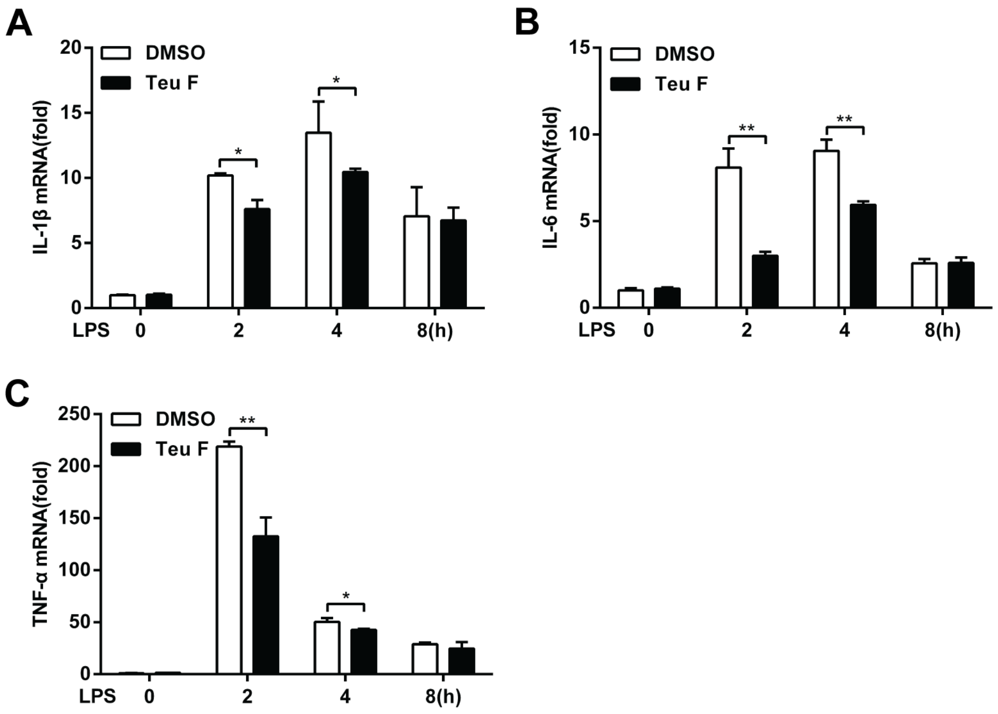

Supplementary Figure S3. Teuvincenone F inhibits LPS-induced proinflammatory cytokine expression in THP1 cells. (**A**-**C**) THP1 cells were pretreated with DMSO or Teuvincenone F (25 μM) for 2 hr, following stimulated with LPS (100 ng/ml) for indicated hours, Q-PCR was used to analyze mRNA expression of IL-1β (**A**), IL-6 (**B**) and TNF-α (**C**). Data are shown as mean ± SD of one representative experiment in (**A**-**C**). *p<0.05, **p<0.01.
