# Supplementary material for: Teuvincenone F Suppresses LPS-Induced Inflammation and NLRP3 Inflammasome Activation by Attenuating NEMO Ubiquitination
Source: Front Pharmacol. 2017 Aug 23;8:565. doi: 10.3389/fphar.2017.00565 (PMC5572209; doi:10.3389/fphar.2017.00565)
Supplement: Supplementary file 4 [file Table1.DOCX]

**Supplementary Table S1:** Q-PCR primers

| Gene name | Forward primer (5’ to 3’) | Reverse primer (5’ to 3’) |
| --- | --- | --- |
| mIL-1β | AACTGTTCCTGAACTCAACTGT | GAGATTTGAAGCTGGATGCTCT |
| mIL-6 | TAGTCCTTCCTACCCCAATTTCC | TTGGTCCTTAGCCACTCCTTC |
| mTNF-α | AAGCCTGTAGCCCACGTCGTA | GGCACCACTAGTTGGTTGTCTTTG |
| mIL-18 | GACAGCCTGTGTTCGAGGAT | TGGATCCATTTCCTCAAAGG |
| mNLRP1a | GGTGGTGTGAAGATGTTGTGT | TCCATGTTCATCGTAGGGACC |
| mNLRP1b | TAGAAACGCCAGATAGGGTGA | AGTGTGATGGAAGTAATGGGGAT |
| mNLRP3 | CGAGACCTCTGGGAAAAAGCT | GCATACCATAGAGGAATGTGATGTACA |
| mNLRC4 | TTGAAGGCGAGTCTGGCAAAG | GGCGCTTCTCAGGTGGATG |
| mNLRP6 | CTCGCTTGCTAGTGACTACAC | AGTGCAAACAGCGTCTCGTT |
| mAIM2 | GTCACCAGTTCCTCAGTTGTG | CACCTCCATTGTCCCTGTTTTAT |
| mβ-actin | AGTGTGACGTTGACATCCGT | GCAGCTCAGTAACAGTCCGC |
| hIL-1β | CAGCTACGAATCTCCGACCAC | GGCAGGGAACCAGCATCTTC |
| hIL-6 | TGCAATAACCACCCCTGACC | GTGCCCATGCTACATTTGCC |
| hTNF-α | CTGGGCAGGTCTACTTTGGG | CTGGAGGCCCCAGTTTGAAT |
| hβ-actin | TGGAGAAAATCTGGCACCACACC | GATGGGCACAGTGTGGGTGACCC |
