# Supplementary material for: Teuvincenone F Suppresses LPS-Induced Inflammation and NLRP3 Inflammasome Activation by Attenuating NEMO Ubiquitination
Source: Front Pharmacol. 2017 Aug 23;8:565. doi: 10.3389/fphar.2017.00565 (PMC5572209; doi:10.3389/fphar.2017.00565)
Supplement: Supplementary file 5 [file Table2.DOCX]

**Supplementary material**

**Supplementary Table S2.** Names of 20 compounds from *P. szemaoensis*.

| No. | Name | No. | Name |
| --- | --- | --- | --- |
| **1** | Polystachyol | **11** | 4-methoxy-lariciresinol-9-​O-*​β*-​D-​  glucopyranoside |
| **2** | 5,4',7-trihydroxy-6-methoxy-  flavone-7-O-*β*-D-  glucopyranoside | **12** | Lariciresinol-9-​O-​*β*-​D-​  glucopyranoside |
| **3** | Homoplantaginin | **13** | Ansecoisolariciresin​ol |
| **4** | Scutellarein-6-​methyl ether | **14** | (3*R*,4*S*)-3,4-Dihydro-6-hydroxy-4-  3-methoxyphenyl)-3-(hydroxyl  methyl)-7-methoxy-2-  naphthalenecar boxaldehyde |
| **5** | 4', 5-dihydroxy-6,7-dimethoxy-  flavone | **15** | Isolariciresinol-9'-​*β*-​D-​  glucopyranoside |
| **6** | 4', 5-dihydroxy-6,7-dimethoxy-  flavanone | **16** | (+)-lyoniresinol |
| **7** | (-​)​-​Lirioresinol B | **17** | Teuvincenone F |
| **8** | Lirioresinol A | **18** | Tortoside F |
| **9** | (+)​-Lirioresinol C | **19** | Erythro-guaiacylglycerol-​*β*-​*O*-​4'-​  coniferyl ether |
| **10** | Palatiferin A | **20** | Threo-​guaiacylglycerol-​*β*-​*O*-​4'-​  coniferyl ether |
